# 

**Published:** 1881-11

**Authors:** 


### Pamphlets.

Medical Communications of the Massachusetts Medical Society.

Sarcoma of the Choroid, Ciliary Body and Iris. By J. S. Prout, M.D., Brooklyn, and Charles S. Bull, M.D., New York. Reprint from the Archives of Ophthalmology.

Uterine Dilatation with a New Instrument. By H. P. C. Wilson, M.D., Baltimore. Reprint from the American Journal of Obstetrics and Diseases of Women and Children.

Convulsions Due to Depression of Spinal Reflex Inhibitory Centres; with Special Reference to the Convulsions of Apomorphine, Atropine, Strychnine and Other Poisons. By Edward T. Reichert, M.D.

Ethylene Bichloride as an Anæsthetic Agent; with a Consideration of Ethylene Methylethylate, Ethylene Ethylate, Ethyl Nitrate, and Ethylidene Bichloride. By Edward T. Reichert, M.D.

Annual Address delivered before the Medical Association of Georgia, at Thomasville, April 20, 1881, by the President, J. C. LeHardy, M.D., Savannah. Reprint from the Transactions of the Medical Association of Georgia.

Female Diseases; the result of errors in habit and hygiene during childhood and puberty; with remarks on the treatment of rachialgia with igni-puncture (Paque lins cautery). By R. J. Nunn, M.D., Savannah. Reprint from the Transactions of the Medical Association of Georgia.

Chronic Pelvic Abscess. A contribution to the differential diagnosis of abdominal tumors. By A. F. Erich, M.D., Baltimore.

Report of Section on Ophthalmology and Otology, Medical and Chirurgical Faculty of Maryland. By Samuel Theobald, M.D., Baltimore. Reprint from Transactions, 1881.

Annual Report of the Board of Health of the City of Indianapolis for the year 1880. E. S. Elder, M.D., President; W. E. Jeffries, M.D., Secretary.

Ovariotomy During Pregnancy. By H. P. C. Wilson, M.D., Baltimore. Reprint from Vol. V, Gynecological Transactions, 1881.

Optic Neuritis. By A. Frieudenwall, M.D., Baltimore. Reprint from Maryland Medical Journal.

Report of the Section on Gynecology for the Seventh Congressional District, to the Medical Association of Georgia. By S. H. Stout, M.D., late of Roswell, now of Chattanooga, Tenn. Reprint from Transactions, 1881.

